# Machine learning models for identifying predictors of clinical outcomes with first-line immune checkpoint inhibitor therapy in advanced non-small cell lung cancer

**DOI:** 10.1038/s41598-022-20061-6

**Published:** 2022-10-21

**Authors:** Ying Li, Matthew Brendel, Ning Wu, Wenzhen Ge, Hao Zhang, Petra Rietschel, Ruben G. W. Quek, Jean-Francois Pouliot, Fei Wang, James Harnett

**Affiliations:** 1 Regeneron Pharmaceuticals, Inc., 777 Old Saw Mill River Road, Tarrytown, NY 10591 USA; 2 Institute for Computational Biomedicine, Department of Physiology and Biophysics, Weill Cornell Medicine, New York, NY USA; 3 Department of Population Health Sciences, Weill Cornell Medicine, New York, NY USA

**Keywords:** Health care, Cancer, Computational science, Risk factors, Outcomes research

## Abstract

Immune checkpoint inhibitors (ICIs) are standard-of-care as first-line (1L) therapy for advanced non-small cell lung cancer (aNSCLC) without actionable oncogenic driver mutations. While clinical trials demonstrated benefits of ICIs over chemotherapy, variation in outcomes across patients has been observed and trial populations may not be representative of clinical practice. Predictive models can help understand heterogeneity of treatment effects, identify predictors of meaningful clinical outcomes, and may inform treatment decisions. We applied machine learning (ML)-based survival models to a real-world cohort of patients with aNSCLC who received 1L ICI therapy extracted from a US-based electronic health record database. Model performance was evaluated using metrics including concordance index (c-index), and we used explainability techniques to identify significant predictors of overall survival (OS) and progression-free survival (PFS). The ML model achieved c-indices of 0.672 and 0.612 for OS and PFS, respectively, and Kaplan–Meier survival curves showed significant differences between low- and high-risk groups for OS and PFS (both log-rank test p < 0.0001). Identified predictors were mostly consistent with the published literature and/or clinical expectations and largely overlapped for OS and PFS; Eastern Cooperative Oncology Group performance status, programmed cell death-ligand 1 expression levels, and serum albumin were among the top 5 predictors for both outcomes. Prospective and independent data set evaluation is required to confirm these results.

## Introduction

Lung cancer is the second most common cancer with an estimated 236,740 new cases expected to be diagnosed in 2022 in the US^1^. More than 80% of lung cancer cases are attributed to non-small cell lung cancer (NSCLC) and most of these cases are diagnosed at advanced stages. Lung cancer continues to be the leading cause of cancer-related death, with 130,180 such deaths expected in 2022 in the US^1^. However, using data from Surveillance, Epidemiology, and End Results (SEER), Howlader et al.^2^ reported a significant reduction in mortality for lung cancer between 2013 and 2016, especially for the NSCLC subtype. This reduction was potentially associated with major advancements in treatment, including the development and approval of targeted therapies and immunotherapies.

Immune checkpoint inhibitors (ICIs) targeting programmed cell death protein 1 (PD-1) or its ligand (PD-L1) have become the standard of care for first-line (1L) treatment of advanced NSCLC (aNSCLC) without actionable oncogenic driver mutations^3^. However, survival benefits compared with chemotherapy were not consistent in randomized trials among all ICIs in aNSCLC^4^. Further, while some trial subpopulations may have better survival, the trials may not have been statistically powered for formal evaluation of these subgroups^4^. Patients from randomized trials may not be fully representative of the population seen in clinical practice, since trial inclusion and exclusion criteria often select a younger population with fewer comorbidities and who are more likely to achieve better outcomes under the care and close supervision requirements of the clinical trial. Hence, a better understanding of how patient outcomes may vary in a real-world population and identifying predictors of outcomes in the clinical practice setting is important when considering treatment decisions.

There has been a substantial increase in the availability of real-world clinical data resulting from the adoption of electronic health records (EHRs). This availability offers a timely and valuable alternative to traditional cohort studies (e.g., registries)^5^, with the potential advantage of efficiently collecting detailed longitudinal measurements of clinical characteristics and patient care in large populations. EHRs can provide real-world treatment and follow-up data, clinical laboratory test results, and increasingly, genetic and molecular profiling data. However, the complexity, diversity, and incompleteness of EHR data may introduce difficulties in discovering novel insights related to clinical outcomes, noting the EHR is designed for clinical practice management rather than research.

The Cox proportional hazard (CPH) model is the most frequently used method in oncology to identify prognostic factors that impact disease progression or patient survival^6,7^. However, as this model assumes that the outcome is a linear combination of covariates, it may not account for complex nonlinear relationships of the covariates nor interactions among covariates. In addition, it is an inadequate model for handling the high dimensional data that may be available from EHRs. In recent years, machine learning (ML)-based approaches have been shown to complement CPH for improving cancer diagnosis, detection, prediction, and prognosis^8^. Several studies have found that ML methods can perform at least as well as CPH in predicting patient survival^8–10^. Moreover, ML explainability technique has emerged as a means to improve the transparency and interpretability of complex ML models, which are increasingly being recognized as essential tools in the healthcare domain. In order to garner trust and then adopt ML models, it is important that healthcare stakeholders understand how ML models arrive at a decision^9^.

SHapley Additive exPlanation values (SHAP) are a type of ML explainability technique^11^. SHAP values describe the importance of a variable when making a prediction for a specific data point, with positive or negative values indicating the direction of the effect. SHAP values have been used in many oncology-related ML studies and have shown promising results^12–14^.

Statistical and machine learning models have been applied to aNSCLC-related clinical trials, registries, and real-world data to predict clinical outcomes, recommend individualized treatment, and identify cancer patients in different stages^15,16^. For example, Siah et al.^16^ used methods including penalized logistic regression, random forest models, and multilayer perceptrons to analyze aggregated patient-level data from 17 randomized clinical trials evaluating molecular-targeted therapy and immunotherapy in patients with aNSCLC. Their study reported that biomarker status (i.e., presence of PD-L1, epidermal growth factor receptor [*EGFR*], or anaplastic lymphoma kinase [*ALK*]) was the strongest predictor of objective response, progression-free survival (PFS), and overall survival (OS). They also found that the performance of predictive models was poorest when applying the predictive models to patients on anti-PD-1/PD-L1 therapy compared with model performance on patients with chemotherapy or targeted therapy. The authors hypothesized that other clinical factors and composite multiomic signatures beyond PD-L1 positivity were at work in response to anti-PD-1/PD-L1 ICI therapy, but such clinical trial data were absent. She et al. applied the DeepSurv approach to SEER data to predict survival and provide individualized treatment recommendations in a population of patients newly diagnosed with Stages I to IV NSCLC between January 2010 and December 2015^17,18^. Yuan et al.^19^ used penalized Cox regression to predict OS and identify significant predictors of survival among patients newly diagnosed with any stage of NSCLC from January 2000 through January 2015 based on structured and unstructured longitudinal EHR data. The unstructured data were processed using natural language processing, and patients were classified as having NSCLC according to an ML-based classification algorithm.

To our knowledge, there is a lack of studies exploring the performance of ML methods with the aim of identifying significant predictors of clinically relevant outcomes in real-world patients with aNSCLC who initiate 1L ICI therapy using point of care data collected in the EHR. We developed ML-based survival models using a large US-based, nationally representative EHR-derived aNSCLC database to predict clinical outcomes including OS and PFS among populations of interest. We compared the performance of multiple ML models using concordance index, hinge loss, hinge loss at 1 year, hinge loss at 2 years, and margin loss. Moreover, we identified significant predictors of clinical outcomes and used SHAP values to facilitate model interpretation based on the ML model with the best performance.

## Methods

### Data source and patient selection

This was a retrospective cohort study of patients with aNSCLC initiating 1L ICI treatment in the Flatiron Health aNSCLC database. The Flatiron Health EHR-derived database is a longitudinal database comprising de-identified patient-level structured and unstructured data curated via technology-enabled abstraction^20,21^. During the study period (2015–2021), the Flatiron Health network consisted of approximately 280 US cancer clinics (~ 800 sites of care). The data are subject to obligations to prevent re-identification and protect patient confidentiality. The institutional review board of WCG IRB, Puyallup, WA, approved the study protocol for data collection from the real-world cohort prior to conduct of the study and waived the need of informed consent.

A cohort of patients was selected who were newly diagnosed with aNSCLC between January 1, 2015, and November 30, 2020, and met the following inclusion and exclusion criteria: age ≥ 18 years at the time of aNSCLC diagnosis, received an ICI(s)-containing regimen as 1L therapy within 90 days after aNSCLC diagnosis (index date = 1L initiation date), had ≥ 1 PD-L1 test on or before the index date, had no positive test results or receipt of targeted therapies for *ALK/EGFR/ROS1/BRAF/KRAS* oncogene alteration(s), and no clinical trial participation.

Two clinical outcomes were evaluated, of which the first was OS, defined as time from the index date to death. Patient-level structured data (EHRs, obituaries, and the Social Security Death Index) and unstructured EHR data (abstracted) were already linked to generate a composite mortality variable that has high sensitivity and specificity when compared to the National Death Index (NDI)^22^. The second outcome was PFS, defined as time from index date to the first real-world progression event or death. Real-world progression was available based on already abstracted information from the medical charts and defined as distinct episodes in the patient journey at which time the treating physician or clinician concluded that there was spread or worsening of the disease. Flatiron Health uses a clinician-anchored approach supported by radiology reports for assessing real-world progression, since this has been reported to be the optimal and most practical method for such assessment^23^.

### Variable pre-processing

Several categories of candidate variables were considered in our models for predicting clinical outcomes, including demographics, medical history, tumor characteristics, comorbidities, metastatic sites, types of 1L treatment, concomitant medications, and laboratory measurements. The assessment time windows, which were determined by clinical experts, varied across variables, and their details are described below and in Table S1.

The demographic variables that were considered included age on the index date (i.e., initiation of 1L therapy), sex, payer type, race, and geographic region. Medical history included year of aNSCLC diagnosis, smoking status, number of different types of medical visits 90 days prior to index date, and the baseline Eastern Cooperative Oncology Group (ECOG) score, which was defined as the most recent value prior to or on the index day or the highest of the values if more than one ECOG score was reported on the same day. Tumor characteristics included diagnosis status, histology, and PD-L1 expression level, which was assessed based on all valid PD-L1 percentage stain results on or before the index date; the highest PD-L1 percentage staining level was abstracted for each patient. Comorbidities, presence of other primary cancer, and site of metastasis were assessed based on all ICD-10 and ICD-9 diagnoses documented on or before the index date. Furthermore, comorbidities based on ICD-10 and ICD-9 codes were summarized using the Elixhauser comorbidity index score and categorized into 29 different groups excluding 2 groups of metastatic cancer and solid tumor without metastasis^24^. The concomitant medications used during the 90 days before or on the index date were grouped by the third level of anatomical therapeutic chemical (ATC3) codes and the number of different kinds of drugs was captured. For example, if a patient was on abacavir (J05AF06), dolutegravir (J05AJ03) and lamivudine (J05AF05), then the ATC3 variable of J05A for this patient was 3. ATC3 codes were removed if the ATC3 class was taken by < 10% of patients. Vital signs and laboratory tests were limited to those most frequently measured among the study population and were assessed in > 50% of patients within 90 days prior to or on the index date. Outliers were defined as lowest and highest 0.1% of the distribution for each assessed laboratory value, and as lowest 10% and highest 0.1% of the distribution for each assessed vital value using empirical analysis; outliers were then set as missing. Missing values were imputed using the following rules: (1) imputed mode value for categorical and binary variables; (2) imputed mean value for most continuous variables; (3) imputed zero for PD-L1 level variable, and then a binary variable was introduced to indicate missingness; (4) imputed zero for metastatic sites and comorbidities. If multiple values were available during the 90-day window prior to or on index date, the frequency of assessments, average value, variation (i.e., standard deviation), and direction and magnitude of changes (i.e., slope) were calculated. Categorical integers were used for initial stage at diagnosis with Stage 0/I = 0, Stage II = 1, Stage IIIA = 2, Stage IIIB/C = 3, and Stage IV = 4. The other categorical variables were one-hot encoded and a category from the same categorical variable was dropped to minimize collinearity. We further excluded a set of features that showed > 85% correlation with the other features as measured using Pearson correlation.

### Models

Survival modeling for time-to-event prediction was necessary due to right-censoring, or drop-out of patients from the cohort prior to event occurrence. Survival modeling requires a set of data in the form $D={\{\left({x}_{i},{\delta }_{i},{t}_{i}\right)\}}_{i=1}^{N}$ where N is the total number of patients in the cohort, ${x}_{i}$ represents the features, ${\delta }_{i}$ represents the indicator variable with ${\delta }_{i}=1$ representing that an event occurred and ${\delta }_{i}=0$ indicating right-censoring, and ${t}_{i}$ is either the time of censoring or time of the event for patient i^25^. We used 5 different approaches to perform time-to-event prediction in the presence of the right-censored data. The predicted median survival time from models was used for the evaluation.

The CPH model is a standard semi-parametric approach that computes the impact of a set of given features on the risk of an event occurring, and assumes the features are independent^26^. We used a penalized Cox regression whereas the regularization parameter and the method to handle tied event times were tuned^27,28^.

The accelerated failure time (AFT) model is a parametric model that can be used as an alternative to CPH models^29^. There are several known distributions that have been used for this model including Weibull, log-normal, log-logistic, and exponential. We used quantile–quantile (QQ) plots to examine which distribution fit our 2 outcomes, and chose the log-logistic AFT model, which considers the relationship between recovery time and covariates as a linear relationship. The rate of false positives and weights of penalization were tuned.

Survival support vector machine (SSVM) used in the study is able to handle right-censored survival data by combining ranking-based and regression-based loss, and its computational efficiency was improved by the use of kernel functions^30^. Weights of penalization, the mixing parameter between ranking and regression loss, and optimizers were tuned.

Gradient-boosted decision tree (GBDT) was used to evaluate whether non-linear relationships identified by increasing model complexity would improve model performance^31^. We used Cox loss for GBDT (GBDT-CPH). Learning rate, number of regression trees, maximum depth of the individual regression estimators, and the fraction of samples to be used for fitting the individual regression estimators were tuned.

DeepSurv is a CPH deep neural network and state-of-the-art survival method that can model increasingly complex relationships between patients’ characteristics and their risk of failure^18^. We used modern deep learning techniques to optimize the training of the network including tuning hyper-parameters of learning rate, dropout rate, number of hidden layers, and number of nodes in each hidden layer.

The related hyperparameters for the above models were tuned using randomized searches of different parameter settings and fivefold cross-validation, and the results were reported based on held-out validation sets from cross-validation, also known as testing set.

### Evaluation

Several metrics were used to quantify how well the model fit the data. First, we used the concordance index (c-index), which measures how well the model ranks patients based on risk score compared to clinical outcomes of interest^32^. Second, we used 2 metrics derived from Haider et al.^33^ called margin loss and hinge loss. Specifically, these are metrics that can handle censored data and quantify model performance in terms of distance of predicted time-to-events and actual time-to-events. Third, since most patients had an event within 2 years post-index, hinge and margin loss scores for patients with an event prior to 1 and 2 years post-index were also reported to reveal whether a model could predict time-to-events accurately.

### Explainability

Understanding how models generate their predictions is important in the clinical domain. We applied 2 different approaches to identify significant predictors. First, model-based importance scores were generated by different models, such as coefficients from Cox regression or tree-based feature importance scores from GBDT. However, a limitation of model-based scores is that some models only report whether a feature is important but do not show the directionality of the association, such as whether higher values lead to higher risk. To address this, we used SHAP values that are model agnostic^11^. Specifically, we used KernelSHAP to assign SHAP values for important variables based on test data.

## Results

### Clinical characteristics

**Figure 1:**
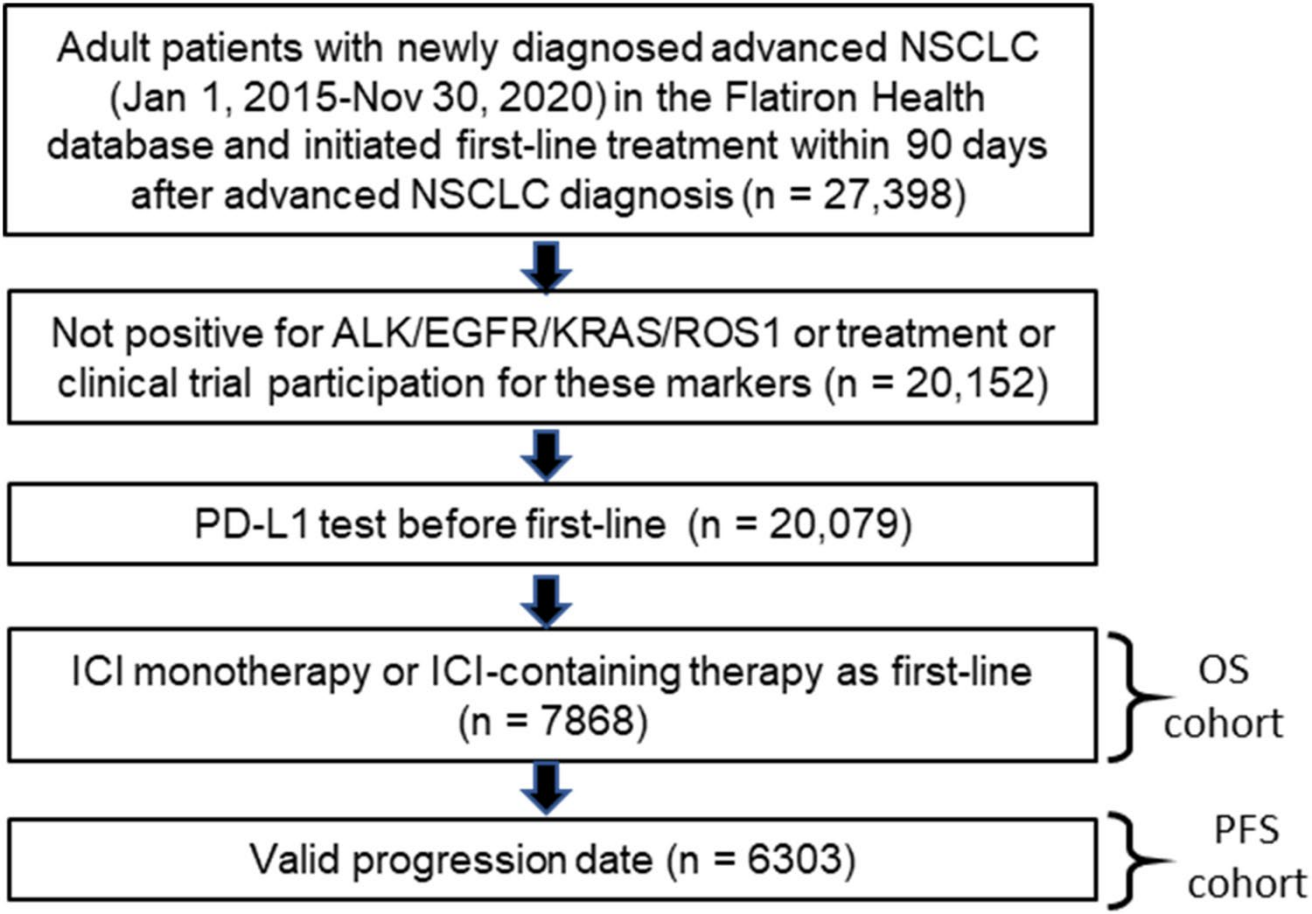
Population attrition after applying inclusion and exclusion criteria. *ALK*, anaplastic lymphoma kinase; *BRAF*, B-Raf proto-oncogene; EHR, electronic health record; ICI, immune checkpoint inhibitor; *KRAS*, Kirsten rat sarcoma virus; NSCLC, non-small cell lung cancer; OS, overall survival; PFS, progression-free survival; *ROS1*, C-ROS oncogene 1.

Figure 1 shows the attrition of the study population after applying inclusion and exclusion criteria. A total of 7868 patients met the study criteria for the OS cohort, of whom 6303 also had real-world progression data and were included in the PFS cohort. As shown in Table 1, the mean follow-up time for the OS cohort was 350.5 days, and 4879 deaths were observed. The mean follow-up time for the PFS cohort was 260.3 days, and 5073 progression events were observed, among whom 3809 died by the time of progression. The demographic and clinical characteristics were similar between the OS and PFS cohorts except for systemic treatment. For systemic treatment, the OS cohort had a lower proportion of patients who received ICI alone and a higher proportion who received ICI in combination with drugs other than platinum-based chemotherapy including anti-VEGF and non-platinum-based chemotherapy (10.9% vs. 0.4% in the PFS cohort).

**Table 1:** Demographic and clinical characteristics of the OS and PFS cohorts.

Variable	OS cohort (n = 7868)	PFS cohort (n = 6303)
Age on index date, years, mean ± SD	69.6 ± 9.4	69.6 ± 9.5
**Sex, n (%)**		
Female	3371 (42.8)	2736 (43.4)
Male	4496 (57.1)	3567 (56.6)
**Race, n (%)**		
Asian	117 (1.5)	92 (1.5)
Black or African American	703 (8.9)	568 (9.0)
Other	731 (9.3)	584 (9.3)
White	5514 (70.1)	4402 (69.8)
Unknown	803 (10.2)	657 (10.4)
**Ethnicity, n (%)**		
Hispanic or Latino	239 (3.0)	194 (3.1)
Non-Hispanic	7629 (97.0)	6109 (96.0)
**Practice type, n (%)**		
Academic	551 (7.0)	476 (7.6)
Community	7317 (93.0)	5827 (92.4)
**Payer, n (%)**		
Commercial	4279 (54.4)	3460 (54.9)
Medicare	1693 (21.5)	1348 (21.4)
Medicaid	129 (1.6)	107 (1.7)
Other/unknown	1767 (22.5)	1388 (22.0)
**Region, n (%)**		
Midwest	1085 (13.8)	854 (13.5)
Northeast	1504 (19.1)	1183 (18.8)
South	3505 (44.5)	2831 (44.9)
West	1090 (13.9)	860 (13.6)
Unknown	684 (8.7)	575 (9.1)
**ECOG performance status before or on the index date, n (%)**		
0	1829 (23.2)	1476 (23.4)
1	2806 (35.7)	2241 (35.6)
2	1140 (14.5)	907 (14.4)
3	246 (3.1)	191 (3.0)
4	13 (0.2)	11 (0.2)
Missing	1834 (23.3)	1477 (23.4)
**Histology, n (%)**		
NSCLC histology NOS	367 (4.7)	288 (4.6)
Non-squamous cell carcinoma	5155 (65.5)	4310 (68.4)
Squamous cell carcinoma	2346 (29.8)	1705 (27.1)
**Smoking status, n (%)**		
History of smoking	7225 (91.8)	5792 (91.9)
No history of smoking	638 (8.1)	507 (8.0)
Unknown/not documented	5 (0.1)	4 (0.1)
**Stage at initial diagnosis, n (%)**		
Stage 0/I	573 (7.3)	459 (7.3)
Stage II	467 (5.9)	298 (4.7)
Stage IIIA	1071 (13.6)	631 (10.0)
Stage IIIB/C	281 (3.6)	231 (3.7)
Stage IV	5264 (66.9)	4546 (72.1)
Unknown	212 (2.7)	138 (2.2)
**Year of initial NSCLC diagnosis**	2017.8 (1.9)	2018.1 (1.7)
**Year of advanced NSCLC diagnosis**	2018.3 (1.3)	2018.5 (1.2)
**Systemic treatment, n (%)**		
ICI only	2685 (34.1)	2685 (42.6)
ICI and chemotherapy	4328 (55.0)	3592 (57.0)
ICI and other	855 (10.9)	26 (0.4)
**Outcome**		
Follow-up days, mean ± SD	350.5 ± 346.4	260.3 ± 297.4
Observed events, n (%)	4879 (62.0)	5073 (80.5)

ECOG, Eastern Cooperative Oncology Group; ICI, immune checkpoint inhibitor; NOS, not otherwise specified; NSCLC, non-small cell lung cancer; OS, overall survival; PFS, progression-free survival; SD, standard deviation.

### Model performance

**Figure 2:**
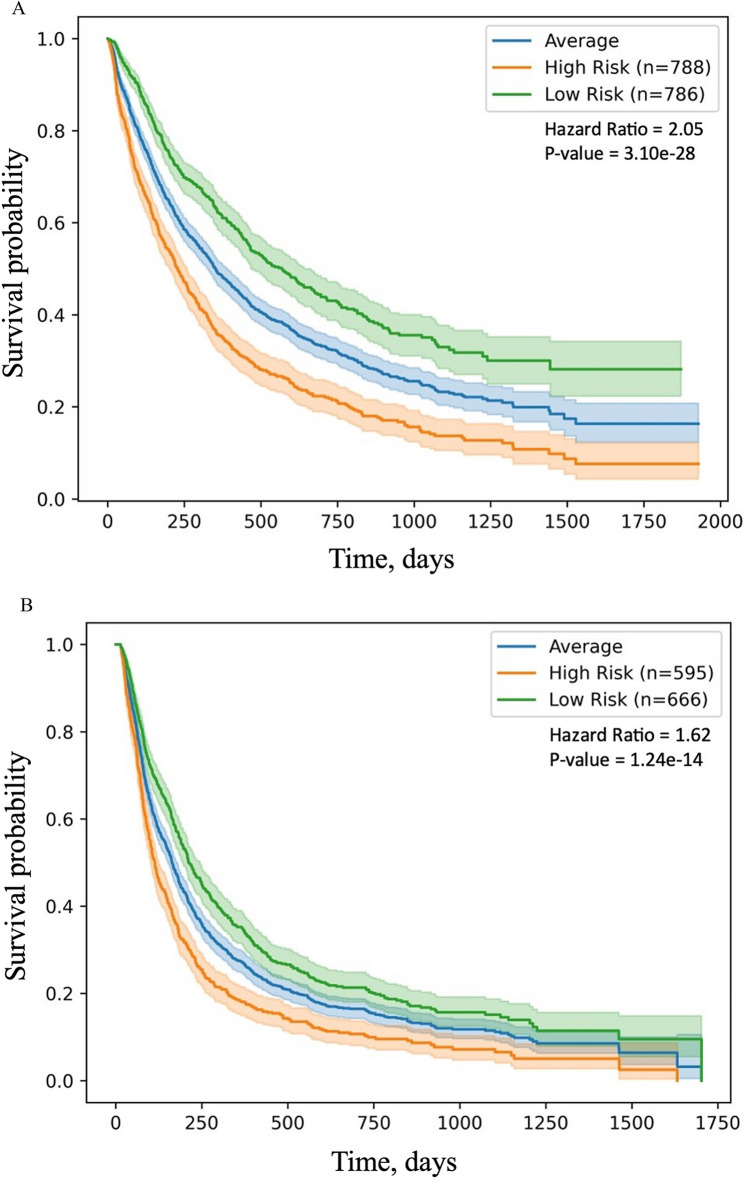
Kaplan–Meier curves for risk stratification. (**A**) Gradient-boosted Cox proportional hazard model for overall survival. (**B**) Gradient-boosted Cox proportional hazard model for progression-free survival. The average curves were generated using observed survival time.

The held-out validation set (also known as testing set) results presented in Table 2a for OS and Table 2b for PFS reflect the best performing models. Each model was parameterized using a randomized search of different parameter combinations with a fivefold cross-validation to maximize the c-index. The parameter space for tuning is reported in Table S2. No single model consistently achieved the best performance. GBDT-CPH had better performance than the other models for OS and PFS on 7 out of 8 performance metrics. The regularized CPH model demonstrated performance similar to GBDT-CPH for the c-index in the testing data set but not for the other metrics. We used the median value of the predicted median survival time derived from the training data to categorize the test data into high- and low-risk groups^34^. The Kaplan–Meier survival curves showed that the difference in both OS and PFS between the low- and high-risk groups was significant (log-rank test p < 0.0001 in Fig. 2), and the hazard ratios (HRs) for OS and PFS were 2.05 and 1.62 between high- and low-risk groups, respectively. The distribution of values for important predictors to differentiate high- and low-risk groups for both OS and PFS are shown in Table S3.

**Table 2:** Comparison of the models for OS and PFS based on held out validation sets.

(a) Overall survival								
Model	Hinge Loss	Margin Loss	Hinge Loss—1 Year	Hinge Loss—2 Year	Hinge Loss—Uncensored	Hinge Loss—Censored	Concordance Index Train	Concordance Index Test
CPH	209.0 (204.2, 213.9)	386.1 (376.6, 395.7)	149.3 (139.5, 159.1)	155.3 (146.2, 164.3)	227.5 (219.5, 235.5)	178.3 (160.5, 196.1)	0.687 (0.685, 0.689)	0.671 (0.660, 0.681)
LogLogisticAFT	212.7 (208.3, 217.1)	387.6 (377.7, 397.5)	153.5 (144.3, 162.8)	161.5 (153.7, 169.4)	234.6 (227.7, 241.5)	**176.4 (160.2, 192.7)**	0.691 (0.689, 0.693)	0.671 (0.662, 0.679)
GBDT (CPH)	**206.8 (199.1, 214.5)**	**384.3 (371.7, 396.9)**	**142.7 (135.6, 149.8)**	**149.3 (142.4, 156.2)**	**219.8 (211.4, 228.1)**	185.2 (170.1, 200.4)	**0.715 (0.712, 0.719)**	**0.672 (0.654, 0.689)**
SSVM	349.5 (338.1, 360.9)	527.6 (508.3, 546.9)	146.2 (142.5, 149.9)	236.0 (234.2, 237.7)	255.4 (242.3, 268.5)	502.5 (486.8, 518.2)	0.692 (0.690, 0.694)	0.671 (0.661, 0.680)
DeepSurv	209.1 (203.3, 215.0)	386.3 (377.0, 395.5)	146.9 (139.1, 154.8)	154.8 (146.0, 163.6)	224.9 (214.6, 235.3)	182.9 (166.1, 199.7)	0.698 (0.691, 0.706)	0.669 (0.656, 0.681)

(b) Progression-free survival								
Model	Hinge Loss	Margin Loss	Hinge Loss—1 Year	Hinge Loss—2 Year	Hinge Loss—Uncensored	Hinge Loss—Censored	Concordance Index Train	Concordance Index Test
CPH	176.7 (171.6, 181.8)	263.8 (253.4, 274.1)	81.5 (79.1, 83.9)	117.3 (114.8, 119.8)	133.0 (128.2, 137.9)	356.1 (327.4, 384.7)	0.635 (0.633, 0.638)	0.611 (0.598, 0.624)
LogLogisticAFT	178.0 (173.7, 182.4)	265.3 (255.7, 274.8)	88.8 (85.0, 92.7)	120.9 (118.0, 123.7)	137.8 (132.7, 142.8)	**343.3 (314.6, 372.1)**	0.642 (0.640, 0.645)	**0.612 (0.597, 0.626)**
GBDT (CPH)	**176.0 (169.9, 182.1)**	**262.9 (252.0, 273.9)**	**77.6 (76.1, 79.1)**	**115.2 (113.2, 117.2)**	**130.6 (127.8, 133.3)**	362.7 (333.7, 391.7)	**0.666 (0.663, 0.668)**	**0.612 (0.602, 0.622)**
SSVM	259.3 (251.3, 267.2)	346.3 (333.4, 359.2)	128.7 (126.1, 131.3)	187.8 (182.4, 193.3)	196.6 (190.3, 202.8)	517.1 (484.1, 550.1)	0.643 (0.640, 0.645)	**0.612 (0.599, 0.625)**
DeepSurv	176.3 (170.7, 182.0)	263.4 (252.7, 274.1)	79.0 (75.6, 82.4)	115.6 (112.8, 118.4)	131.0 (127.3, 134.7)	362.5 (330.8, 394.2)	0.652 (0.650, 0.653)	**0.612 (0.598, 0.625)**

AFT, accelerated failure time; CPH, Cox proportional hazard; GBDT, gradient-boosted decision tree; SSVM, survival support vector machine.

### Explainability of GBDT-CPH model

**Figure 3:**
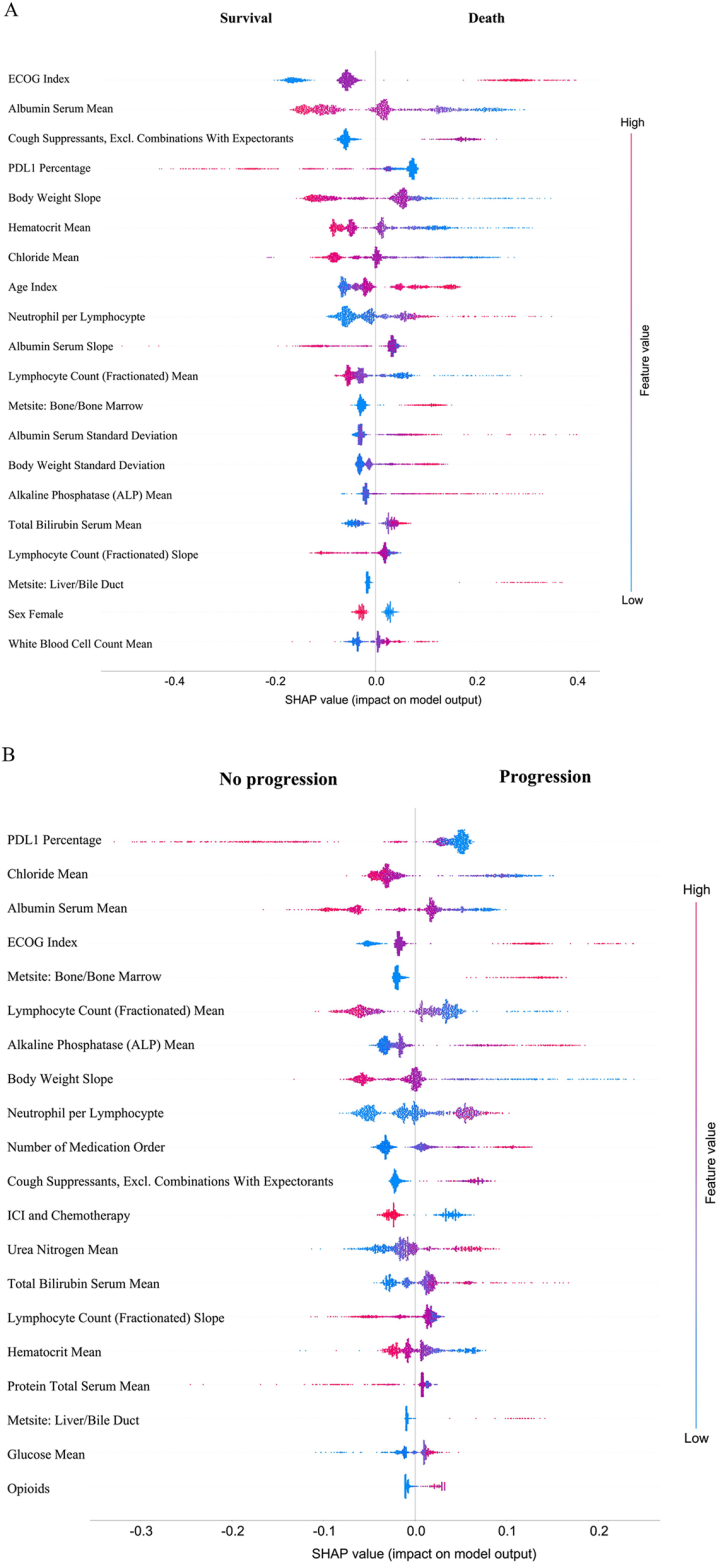
Summary plots for SHAP values. (**A**) Overall survival. (**B**) Progression-free survival. For each predictor, one point corresponds to a single patient, and the x-axis represents the impact of the feature on the model’s output for the specific patient. A positive SHAP value contributes to death or disease progression, while a negative value contributes to OS or PFS. Predictors are arranged along the y-axis based on their ranking: the higher the feature is positioned in the plot, the more significant it is in the model. ECOG, Eastern Cooperative Oncology Group; ICI, immune checkpoint inhibitor; PD-L1, programmed cell death-ligand 1; SHAP, SHapley Additive exPlanation.

As shown in Tables 3 and 4, we extracted the top 20 significant predictors from the GBDT-CPH model and the SHAP values for predicting OS and PFS. Many of these predictors were common between the models, including 18 out of 20 between GBDT-CPH and SHAP for OS (Table 3), and 17 of 20 between GBDT-CPH and SHAP for PFS (Table 4). The R^2^ between OS and PFS was 0.408 (Fig. S1). The summary plots of the SHAP values for the top 20 most significant predictors of OS and PFS are shown in Fig. 3A and B, respectively. Furthermore, the Kaplan–Meier curves, HRs, and p values are reported for the top 20 predictors of OS and PFS in Fig. S2.

**Table 3:** Top 20 predictors of overall survival identified by the gradient-boosted Cox proportional hazard model and SHAP values.

Gradient-boosted Cox proportional hazard model	SHAP
ECOG performance status^38^	*ECOG performance status*^38^
Albumin serum mean^39^	**Albumin serum mean**^39^
Body weight slope^38^	*Cough suppressants, excluding combinations with expectorants*
PD-L1 percentage^38^	**PD-L1 percentage**^38^
Hematocrit mean^41^	**Body weight slope**^38^
Cough suppressants, excluding combinations with expectorants	**Hematocrit mean**^41^
Chloride mean^41^	**Chloride mean**^41^
Neutrophil per lymphocyte^38^	*Age at index*^4^
Lymphocyte count (fractionated) mean^38^	*Neutrophil per lymphocyte*^38^
Alkaline phosphatase (ALP) mean	**Albumin serum slope**^39﻿^
Age at index^4^	**Lymphocyte count (fractionated) mean**^38^
Urea nitrogen mean	*Metastasis site: bone/bone marrow*
Albumin serum standard deviation^39^	*Albumin serum standard deviation*^39^
Albumin serum slope^39^	*Body weight standard deviation*^38^
White blood cell count mean	*Alkaline phosphatase (ALP) mean*^39^
Metastasis site: liver/bile duct	*Total bilirubin serum mean*
Body weight standard deviation^38^	**Lymphocyte count (fractionated) slope**^38^
Metastasis site: bone/bone marrow	*Metastasis site: liver/bile duct*
Lymphocyte count (fractionated) slope^38^	**Sex female**
Monocyte count mean	*White blood cell count mean*

SHAP indicates directionality of the importance (bold: higher values indicate better overall survival; italics: lower values indicate better overall survival).

ECOG, Eastern Cooperative Oncology Group; PD-L1, programmed cell death-ligand 1; SHAP, SHapley Additive exPlanation.

**Table 4:** Top 20 predictors of progression-free survival identified by the gradient-boosted Cox proportional hazard model and SHAP values.

Gradient-boosted Cox proportional hazard model	SHAP
PD-L1 percentage^38^	**PD-L1 percentage**^38^
Body weight slope^38^	**Chloride mean**^41^
Lymphocyte count (fractionated) mean^38^	**Albumin serum mean**^39^
ECOG performance status^38^	*ECOG performance status*^38^
Albumin serum mean^39^	*Metastasis site: bone/bone marrow*
Alkaline phosphatase (ALP) mean^39^	**Lymphocyte count (fractionated) mean**^38^
Chloride mean^41^	*Alkaline phosphatase (ALP) mean*^39^
Metastasis site: bone/bone marrow	**Body weight slope**^38^
Neutrophil per lymphocypte^38^	*Neutrophil per lymphocypte*^38^
Number of medication order	*Number of medication order*
Urea nitrogen mean	*Cough suppressants, excluding combinations with expectorants*
Cough suppressants, excluding combinations with expectorants	**ICI and chemotherapy**^42–44^
Hematocrit mean^41^	*Urea nitrogen mean*
Lymphocyte count (fractionated) slope^38^	*Total bilirubin serum mean*
Protein total serum mean	**Lymphocyte count (fractionated) slope**^38^
Lymphocyte count (fractionated) standard deviation	**Hematocrit mean**^41^
Body weight standard deviation^38^	**Protein total serum mean**
Total bilirubin serum mean	*Metastasis site: liver/bile duct*
ICI and chemotherapy^42–44^	*Glucose mean*
Albumin serum slope	*Opioids*

SHAP indicates directionality of the importance (bold: higher values indicative of better progression-free survival; italics: lower values indicative of better progression-free survival).

ECOG, Eastern Cooperative Oncology Group; PD-L1, programmed cell death-ligand 1; SHAP, SHapley Additive exPlanation.

## Discussion

We developed predictive models for OS and PFS in patients with aNSCLC receiving 1L ICI-containing regimens using regularized CPH and AFT models, and 3 different ML-based models including survival SVM, GBDT-CPH, and DeepSurv. We compared model performance using the c-index, hinge loss, hinge loss at 1 year, hinge loss at 2 years, and margin loss. The results showed that ML-based models can perform as well as or better than the regularized CPH approach on survival prediction tasks shown in Table 2. Summary plots of SHAP values for regularized CPH model were shown in Fig. S3. The regularized CPH model assumes that variables are completely independent and can only capture linear relationships between the variables and the clinical outcomes. This is unlikely the case for cancer progression, as prior literature reported that the interaction effect of different patient characteristics, demographics, and treatment choices would increase or decrease OS or progression time^35,36^. Therefore, ML models that can consider non-linear relationships may be preferable. Our GBDT-CPH model achieved a c-index of 0.672 (95% CI, 0.654–0.689) and 0.612 (95% CI, 0.597–0.626) for OS and PFS, respectively. These findings are consistent with other results reported for ML applications in oncology, although those studies were based on different data sets and patient cohorts. For example, Siah et al.^16^ reported a c-index of 0.561 (95% CI, 0.516, 0.603) for PFS based on aNSCLC patients who were PD-L1 positive and receiving anti-PD-1/PD-L1 ICI therapy in a clinical trial. Similarly, She et al.^17^ reported a c-index of 0.739 (95% CI, 0.713–0.764) for OS based on newly diagnosed NSCLC patients in SEER, and Yuan et al.^19^ reported a c-index of 0.726 for OS based on newly diagnosed NSCLC patients using structured and unstructured EHR data. The patient populations in the latter 2 studies were more heterogeneous than in our study since they used broader inclusion criteria (e.g., not limited to aNSCLC and a particular type of systemic treatment), which is easier to differentiate in terms of survival. It is worth mentioning that DeepSurv did not outperform any of the other methods for 7 metrics predicting OS and PFS. Shwartz-Ziv et al.^37^ compared neural network-based methods versus XGBoost for multiple different tabular data sets, and they found that deep learning models based on tabular data did not outperform XGBoost.

We found that the predictors identified by the GBDT and SHAP values highly overlapped with 18 and 17 of the top 20 predictors that were common for OS and PFS, respectively (Tables 3 and 4). This overlap indicates that SHAP value-based variable importance is consistent with the implicit variable importance scores derived by GBDT-CPH. The most significant predictors of OS were ECOG performance status at index, mean serum albumin, use of cough suppressants, PD-L1 percentage, and body weight slope, while for PFS, PD-L1 percentage, mean chloride, mean serum albumin, ECOG performance status at index, and bone/bone marrow metastases were most significant. It is also worth noting that some predictors were related to different aspects of the same clinical characteristics such as body weight slope and body weight standard deviation, and mean lymphocyte count (fractionated) and lymphocyte count (fractionated) slope.

The SHAP technique can potentially provide a personalized interpretation regarding each patient’s risk of progression and/or death. A higher SHAP value represents a greater contribution to predicted patient outcomes. Figure 3 shows that predictors tend to have long tails in both the OS and PFS models. For example, ECOG performance status score at index has long right tails, indicating the same high ECOG value may contribute to high but different chance of death or progression for individual patients, which intuitively makes sense. Similarly, PD-L1 percentage has long left tails, indicating that similarly high percentages of PD-L1 expression contribute to low but different chance of death or progression for each patient.

Most of the top 20 predictors of OS are supported by the literature^4^. Patients who are younger, have higher PD-L1 expression levels, and have lower ECOG performance status are known to have better outcomes with ICIs^38^. A high neutrophil-to-lymphocyte ratio (NLR) has been associated with poorer OS and is hypothesized to reflect inflammation caused by the tumor and associated increased neutrophil infiltration that promotes cancer progression^38^. Further, the potential protective effect of a higher body weight has been reported and may be attributed to the white adipose tissue involved in the host defense and inflammatory response that may increase sensitivity to anti-PD-1/PD-L1 therapies^4,38^. Decreased albumin is often associated with systemic inflammatory responses and reflects a poor nutritional status, while elevated alkaline phosphatase (ALP) levels are often found when cancer extends to the bone or liver^39^. Both decreased albumin and elevated ALP levels have been independently associated with cancer progression, and more recent research has demonstrated the potential role of the albumin-to-ALP ratio (AAPR) as a prognostic factor in NSCLC and small cell lung cancer, with decreasing AAPR associated with poorer survival^40^. Limited information is available on the potential protective effects of higher chloride and hematocrit levels, and although elevated chloride has been reported to benefit survival, the rationale was not clearly identified^41^. As cough is a common and early symptom of NSCLC that is often captured as a patient-reported outcome in clinical trials, one may expect that lower cough suppressant use is associated with better clinical outcomes, although this association has not been well documented.

The predictors identified for OS and PFS were similar, and 14 of 20 predictors identified by SHAP values were common between OS and PFS. The ECOG performance status score at index, PD-L1 percentage, and mean serum albumin were among the top 5 predictors for both OS and PFS. As PFS is often a surrogate endpoint for OS in cancer trials, it is not surprising that there was substantial overlap between predictors of PFS and OS and, therefore, similar clinical interpretation of the above OS predictors may be applied to those for PFS. Interestingly, medication use including ICI + chemotherapy, number of medication orders, and opioid use were among the top 20 PFS predictors but not OS predictors. The latter 2 variables are likely indicators of comorbidity and management of pain symptoms due to metastases in more advanced cases, and thus, lower values of these variables are associated with better PFS as indicated by SHAP values. Use of chemotherapy with ICI has been shown to provide significant benefits over chemotherapy alone in randomized trials supporting FDA approvals for patients regardless of PD-L1 status^42–44^. Network meta-analyses also suggest that ICI + chemotherapy may offer advantages over ICI monotherapy in OS and PFS^45,46^, hence, the association of ICI + chemotherapy with better PFS in our study appears to be consistent with the literature. However, it is unclear why this was not observed for OS; ICI + chemotherapy was ranked 82nd in significance for OS prediction and should be further evaluated. In addition, the frail patients tended to be treated with ICI monotherapy. It is also interesting to note that age and sex were among the top 20 predictors for OS, in contrast to PFS. These findings are consistent with those from a meta-analysis of 6 randomized trials comparing first-line ICI + chemotherapy versus chemotherapy alone in which the authors reported only PD-L1 expression level (and neither age nor sex) was a significant predictor of PFS treatment benefit while younger age and female sex (as well as nonsquamous vs. squamous histology and anti-PD1 vs. anti-PD-L1 antibody therapy) were associated with greater OS benefit with ICI + chemotherapy^47^. In contrast, Fig. S3 shows that among the top 20 predictors of regularized CPH for OS, being on a Medicare or commercial insurance plan and early stage (Stage I, II) at initial diagnosis but not at the index date were associated with longer survival. Similarly, among the top 20 predictors of regularized CPH for PFS, missing PD-L1 value, being on a Medicare plan, and diagnosed as early stage (Stage I, II) of aNSCLC at initial diagnosis but not at the index date were associated with better PFS.

Our study has several limitations. First, since this study utilized a single, large US-based EHR data set where most of the data are contributed by community oncology practices, the results may not be generalizable to other practice settings. Second, EHR data are designed for clinical documentation rather than research, thus, missing data such as for ECOG performance status is not uncommon and misclassification is possible. Third, since this study did not extract all information from unstructured EHR data, comorbidities were based on diagnosis codes that may have been under- or mis-coded and not fully reflect the patient’s comorbidity status without further evaluation of physician notes. Fourth, the assessment window for candidate variables, the methods to exclude variables highly correlated with other variables, and exclusion of variables with high volume of missing values were not based on established criteria. The potential biases can be analyzed by introducing sensitivity analysis in future studies. Validation in other clinical data sets linked to a variety of other data types including genomic, patient reported, socioeconomic, etc. and ideally in a prospective study will confirm the generalizability of our findings.

## Conclusions

We developed a GBDT-CPH model in combination with explainability techniques to identify predictors of OS and PFS among patients with aNSCLC who used ICI-containing regimens as 1L therapy. The GBDT-CPH model improved performance relative to traditional survival models for both OS and PFS. Predictors identified by GBDT-CPH and SHAP significantly overlapped for OS and PFS. Furthermore, we demonstrated that the identified predictors are mostly consistent with the published literature and/or clinical expectations. These results confirm that the application of ML models in combination with EHR data can provide insight regarding clinical outcomes among aNSCLC patients treated with 1L ICIs.

## Supplementary Information

Supplementary Figures.

Supplementary Tables.

## Data Availability

The data that support the findings of this study have been originated by Flatiron Health, Inc. and are not publicly available, in order to safeguard the terms that ensure that the data remain de-identified. These de-identified data may be made available upon request and are subject to a license agreement with Flatiron Health; interested researchers should contact DataAccess@flatiron.com to determine licensing terms.
