# Supplementary material for: Machine learning models for identifying predictors of clinical outcomes with first-line immune checkpoint inhibitor therapy in advanced non-small cell lung cancer
Source: Sci Rep. 2022 Oct 21;12:17670. doi: 10.1038/s41598-022-20061-6 (PMC9586943; doi:10.1038/s41598-022-20061-6)

**Figure S1.** Correlation between progression-free survival and overall survival. (A) Scatter plot between overall survival and progression-free survival using all available data – patients had both overall survival and progression-free survival outcomes recorded. (B) Segmentation of the scatter plot into four categories: OS0_PFS0, patients did not progress or die; OS0_PFS1, patients progressed but did not die; OS1_PFS0, patients died without progressing (only 15 patients belonged to this category, which could be due to different data delivery, since overall survival data were delivered on March 2021 and progression-free survival data were delivered on November 2021); OS1_PFS1, patients progressed and died. OS, overall survival; PFS, progression-free survival.

A


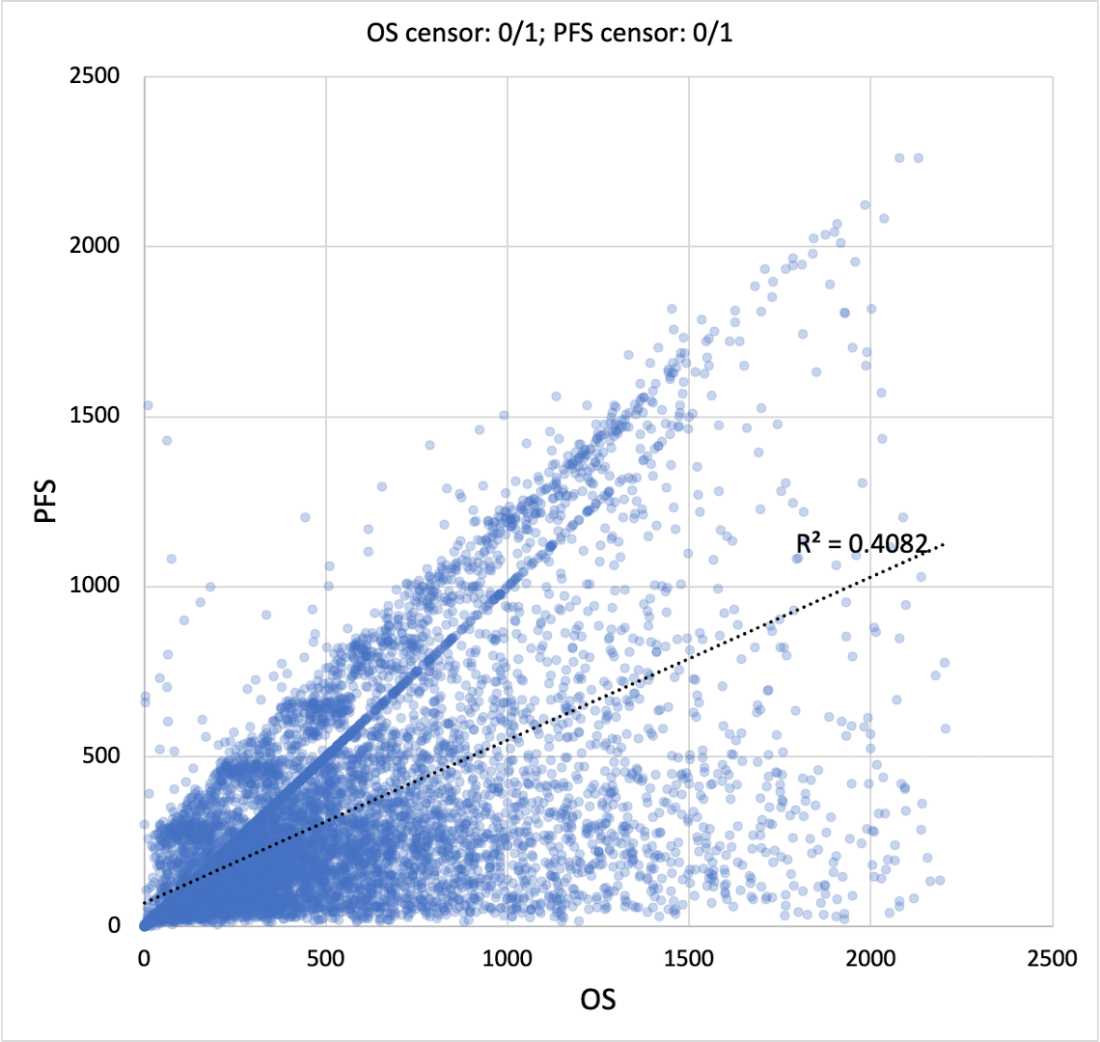


B


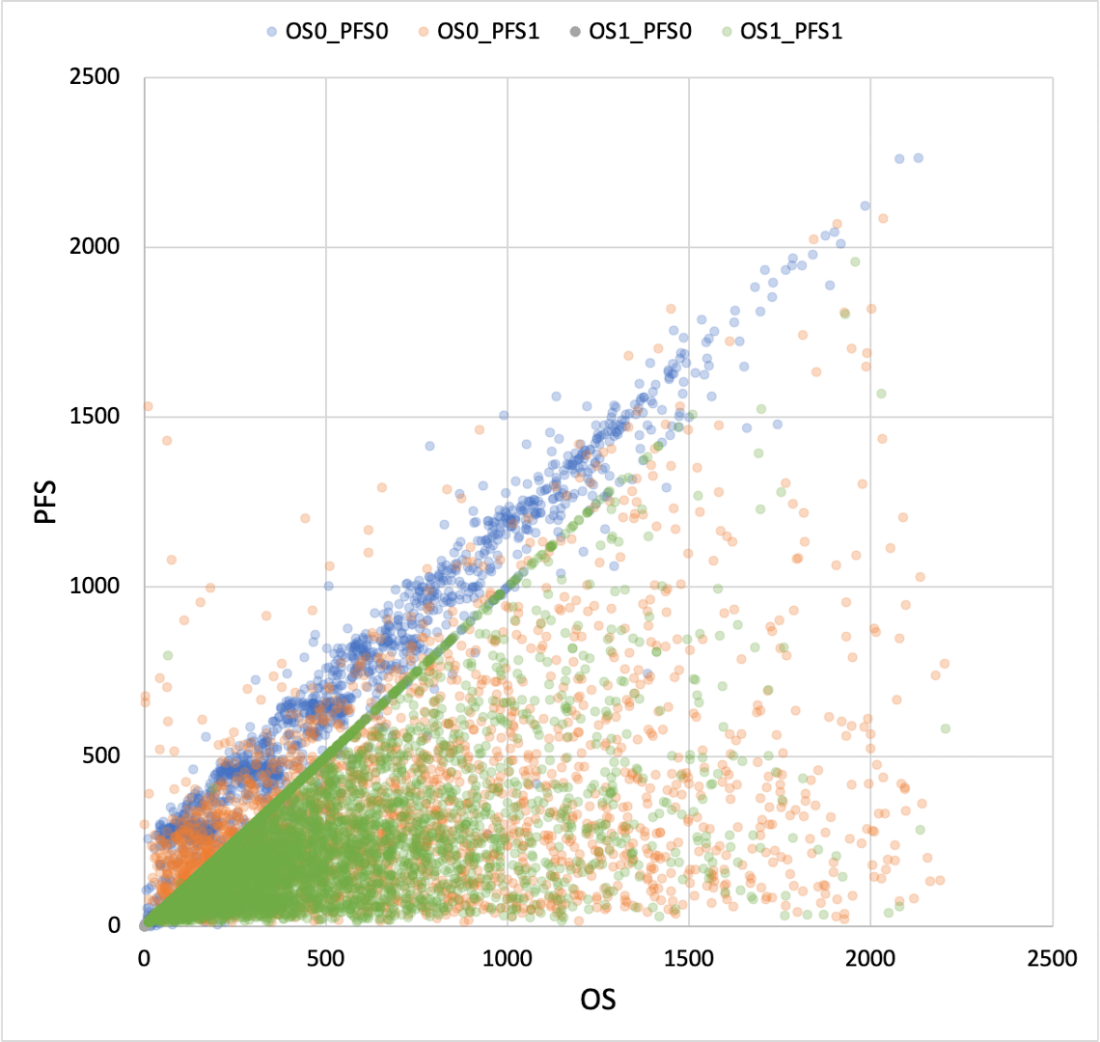


Figure S2 Post hoc analysis of KM plots, hazard ratios and p-values for top 20 predictors identified by GBDT-CPH and SHAP values. Binary predictor’s value of 1 is categorized as high value group and of 0 is categorized as low value group. Categorical and continuous predictors are categorized into high value and low value groups according to their median value whereas values >= median value is categorized as high value group and values < median value is categorized as low value group. (A) Overall Survival and (B) Progression Free Survival. Red in SHAP plot: higher values of the predictors; blue in SHAP plot: lower values of predictors.

1. Overall Survival

For example, lower ECOG at index time (blue) is indicative of survival and higher ECOG at index time (red) is indicative of death, which are mapped to low value and high value categories respectively in the above KM plot


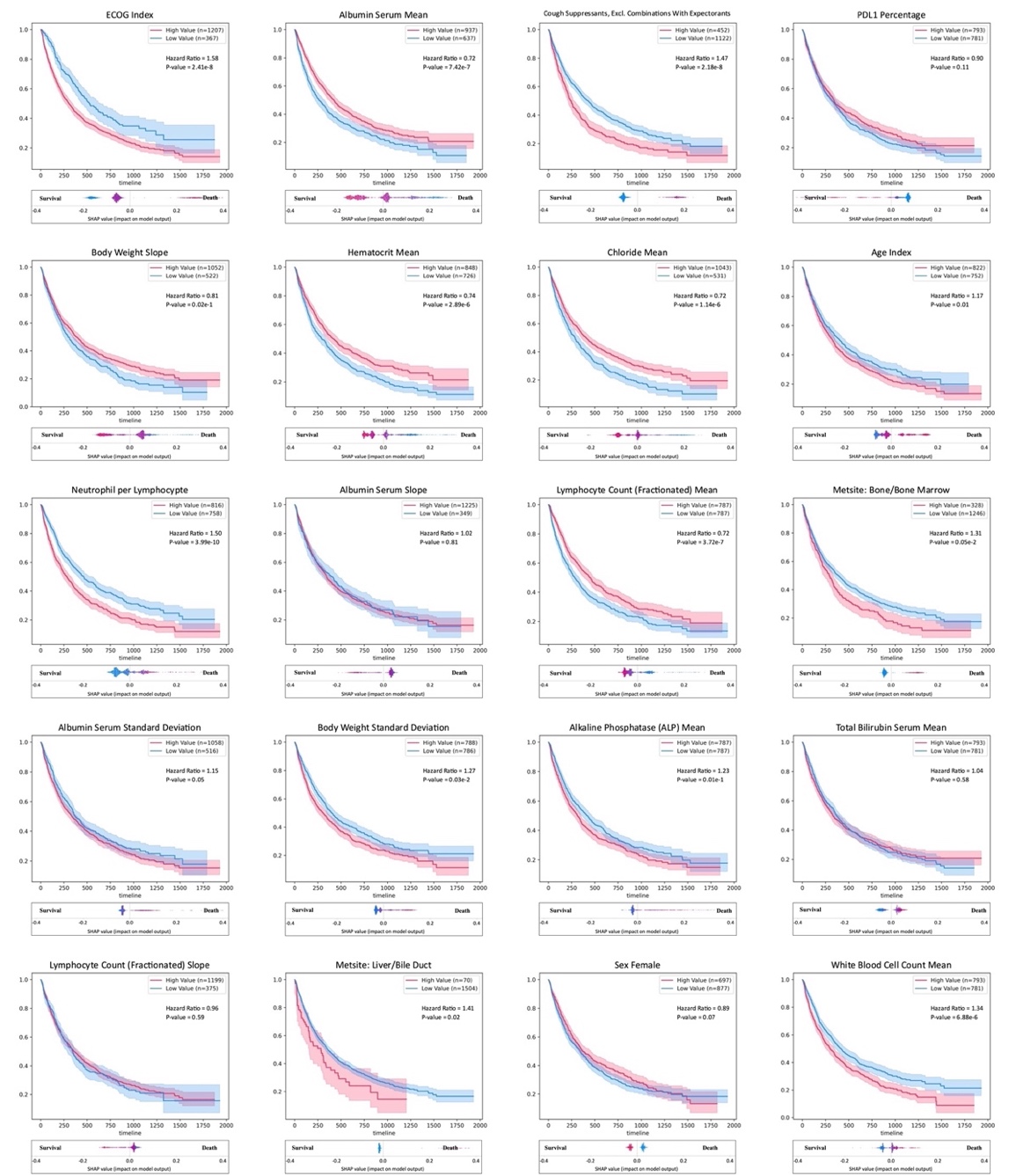


1. Progression Free Survival

For example, higher PD-L1 level (red) in SHAP plot is indicative of no progression and lower PD-L1 level (blue) is indicative of progression, which are mapped to high value and low value categories respectively in the above KM plot.


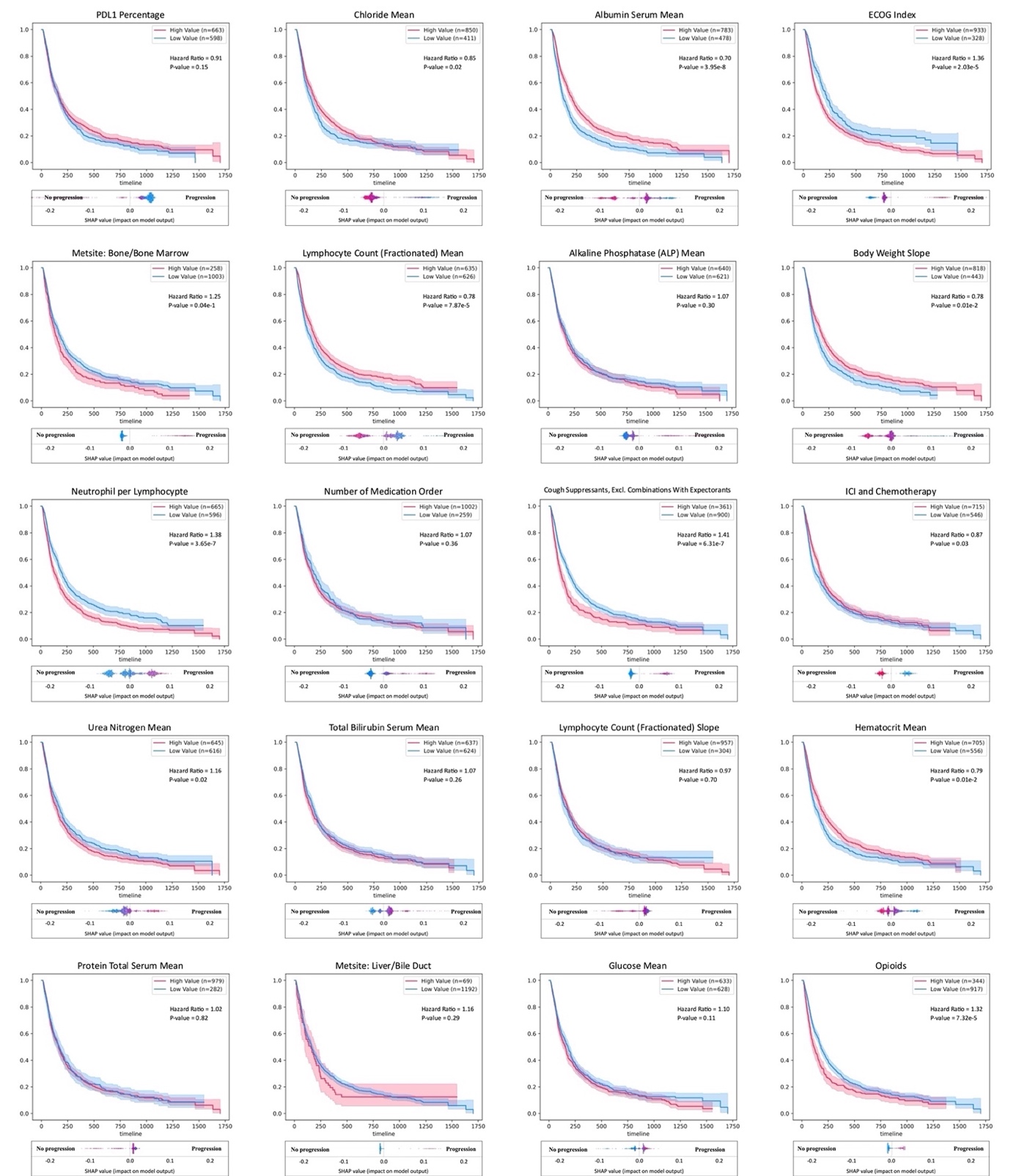
**Figure S3.** Summary plots for SHAP values based on regularized CPH model. (A) Overall survival. (B) Progression-free survival. For each predictor, one point corresponds to a single patient, and the x-axis represents the impact of the feature on the model’s output for the specific patient. A positive SHAP value contributes to death or disease progression, while a negative value contributes to OS or PFS. Predictors are arranged along the y-axis based on their ranking, the higher the feature is positioned in the plot, the more significant it is in the model. ECOG, Eastern Cooperative Oncology Group; ICI, immune checkpoint inhibitor; PD-L1, programmed cell death-ligand 1.

A


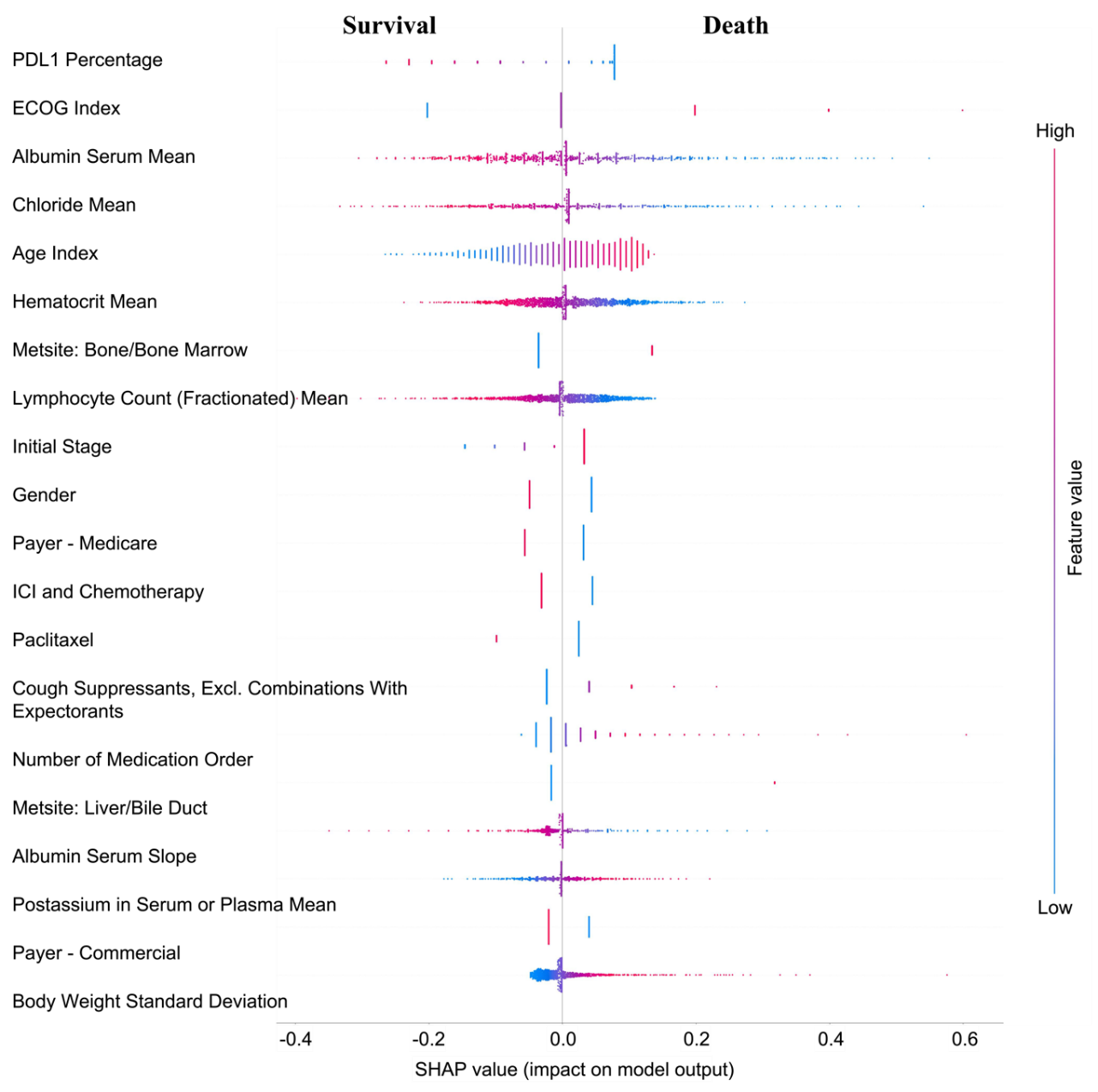


B


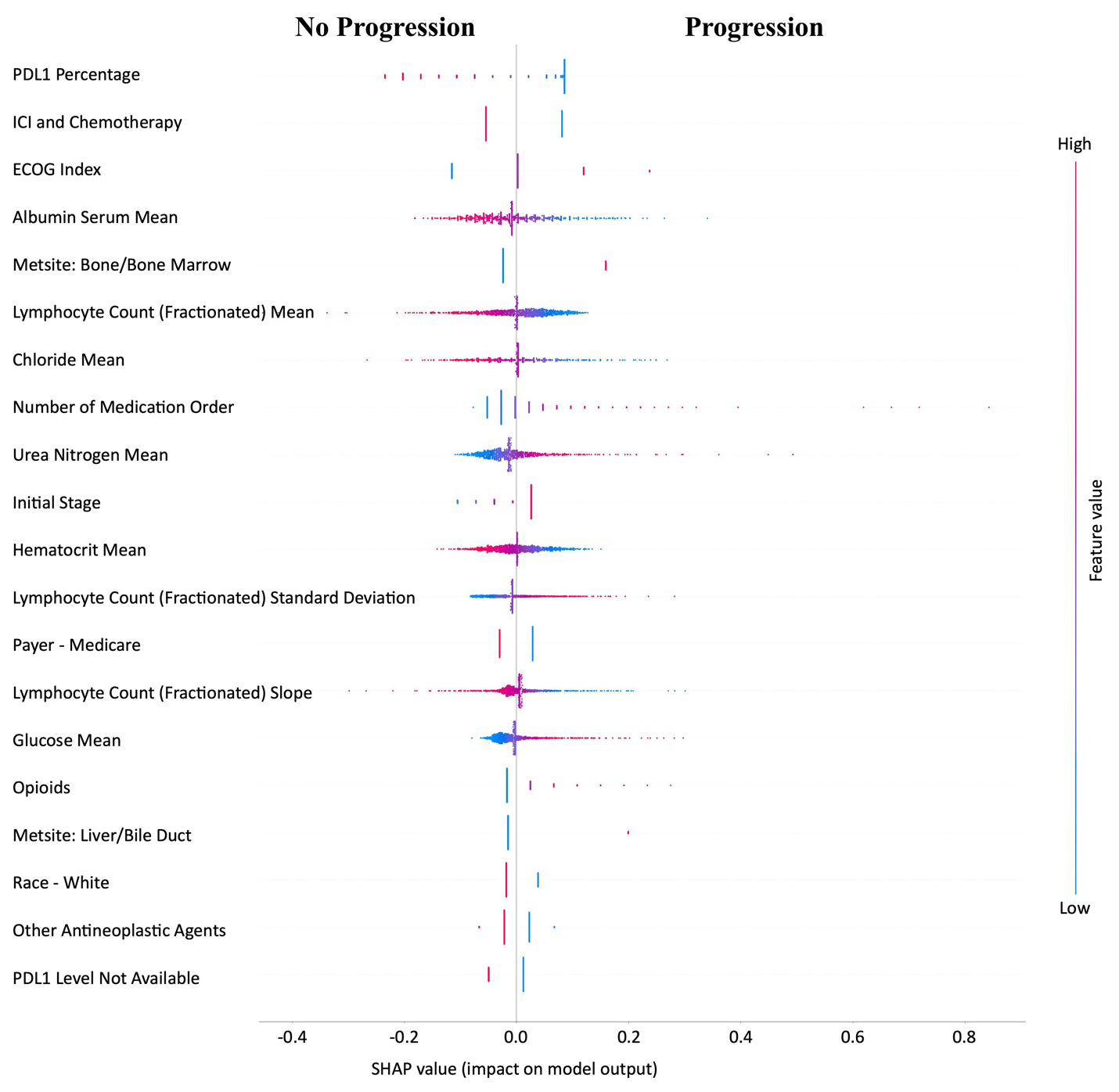

Supplement: Supplementary file 1 — Supplementary Figures. [file 41598_2022_20061_MOESM1_ESM.docx]
