# Supplementary material for: Machine learning models for identifying predictors of clinical outcomes with first-line immune checkpoint inhibitor therapy in advanced non-small cell lung cancer
Source: Sci Rep. 2022 Oct 21;12:17670. doi: 10.1038/s41598-022-20061-6 (PMC9586943; doi:10.1038/s41598-022-20061-6)
Supplement: Supplementary file 2 — Supplementary Tables. [file 41598_2022_20061_MOESM2_ESM.docx]

**Table S1.** Candidate predictors considered in the overall dataset

| **Type** | **Candidate predictor** | **Description** | **Time frame** |
| --- | --- | --- | --- |
| Tumor | denovo | Diagnosis status | [–∞, 0] |
| Tumor | histology_NS | Histology without squamous | [–∞, 0] |
| Tumor | histology_S | Histology with squamous | [–∞, 0] |
| Tumor | ihcclone_22C3 | Immunohistochemistry clone 22C3 | [–∞, 0] |
| Tumor | ihcclone_28-8 | Immunohistochemistry clone 28-8 | [–∞, 0] |
| Tumor | ihcclone_E1L3N | Immunohistochemistry clone E1L3N | [–∞, 0] |
| Tumor | ihcclone_SP142 | Immunohistochemistry clone SP142 | [–∞, 0] |
| Tumor | ihcclone_SP263 | Immunohistochemistry clone SP263 | [–∞, 0] |
| Tumor | Initial stage | Stage at initial diagnosis | [–∞, 0] |
| Tumor | PDL1_Missing | If a PD-L1 value is missing for a patient | [–∞, 0] |
| Tumor | PDL1_pct | PD-L1 percentage | [–∞, 0] |
| Trt | Atezolizumab | Atezolizumab | LOT1 duration |
| Trt | Bevacizumab | Bevacizumab | LOT1 duration |
| Trt | Carboplatin | Carboplatin | LOT1 duration |
| Trt | Cisplatin | Cisplatin | LOT1 duration |
| Trt | Docetaxel | Docetaxel | LOT1 duration |
| Trt | Durvalumab | Durvalumab | LOT1 duration |
| Trt | Etoposide | Etoposide | LOT1 duration |
| Trt | Gemcitabine | Gemcitabine | LOT1 duration |
| Trt | Ipilimumab | Ipilimumab | LOT1 duration |
| Trt | LOT1chemo | LOT1 includes chemotherapy | LOT1 duration |
| Trt | Nivolumab | Nivolumab | LOT1 duration |
| Trt | numdrugs1L | Number of drugs in LOT1 | LOT1 duration |
| Trt | Pembrolizumab | Pembrolizumab | LOT1 duration |
| Trt | Pemetrexed | Pemetrexed | LOT1 duration |
| Trt | Paclitaxel | Paclitaxel | LOT1 duration |
| Trt | Carboplatin | Carboplatin | LOT1 duration |
| Meta | metsite1 | Metastasis site: lung | [–∞, 0] |
| Meta | metsite10 | Metastasis site: kidney | [–∞, 0] |
| Meta | metsite11 | Metastasis site: bladder | [–∞, 0] |
| Meta | metsite12 | Metastasis site: skin | [–∞, 0] |
| Meta | metsite13 | Metastasis site: brain/cerebral meninges | [–∞, 0] |
| Meta | metsite14 | Metastasis site: other CNS | [–∞, 0] |
| Meta | metsite15 | Metastasis site: bone/bone marrow | [–∞, 0] |
| Meta | metsite16 | Metastasis site: ovary | [–∞, 0] |
| Meta | metsite17 | Metastasis site: adrenal gland | [–∞, 0] |
| Meta | metsite18 | Metastasis site: unspecified | [–∞, 0] |
| Meta | metsite19 | Metastasis site: lymph nodes | [–∞, 0] |
| Meta | metsite2 | Metastasis site: mediastinum | [–∞, 0] |
| Meta | metsite3 | Metastasis site: pleura | [–∞, 0] |
| Meta | metsite4 | Metastasis site: other respiratory | [–∞, 0] |
| Meta | metsite5 | Metastasis site: small intestine | [–∞, 0] |
| Meta | metsite6 | Metastasis site: large intestine/rectum | [–∞, 0] |
| Meta | metsite7 | Metastasis site: retroperitoneum/peritoneum | [–∞, 0] |
| Meta | metsite8 | Metastasis site: liver/bile duct | [–∞, 0] |
| Meta | metsite9 | Metastasis site: other digestive organs | [–∞, 0] |
| Vital | Body Weight_count | How many times the body weight was measured | [–90, 0] |
| Vital | Body Weight_mean | Average value of body weight | [–90, 0] |
| Vital | Body Weight_slope | Trend of body weight | [–90, 0] |
| Vital | Body Weight_std | Variation of body weight | [–90, 0] |
| Lab | Neutrophil per Lymphocyte | Neutrophil to lymphocyte ratio | [–90, 0] |
| Lab | Alanine aminotransferase (ALT or SGPT)_mean | Average value of alanine aminotransferase (ALT or SGPT) | [–90, 0] |
| Lab | Alanine aminotransferase (ALT or SGPT)_slope | Trend of alanine aminotransferase (ALT or SGPT) | [–90, 0] |
| Lab | Alanine aminotransferase (ALT or SGPT)_std | Variation of alanine aminotransferase (ALT or SGPT) | [–90, 0] |
| Lab | Albumin, serum_mean | Average value of albumin, serum | [–90, 0] |
| Lab | Albumin, serum_slope | Trend of albumin, serum | [–90, 0] |
| Lab | Albumin, serum_std | Variation of albumin, serum | [–90, 0] |
| Lab | Alkaline phosphatase (ALP)_mean | Average value of alkaline phosphatase (ALP) | [–90, 0] |
| Lab | Alkaline phosphatase (ALP)_slope | Trend of alkaline phosphatase (ALP) | [–90, 0] |
| Lab | Alkaline phosphatase (ALP)_std | Variation of alkaline phosphatase (ALP) | [–90, 0] |
| Lab | Aspartate aminotransferase (AST or SGOT)_mean | Average value of aspartate aminotransferase (AST or SGOT) | [–90, 0] |
| Lab | Aspartate aminotransferase (AST or SGOT)_slope | Trend of aspartate aminotransferase (AST or SGOT) | [–90, 0] |
| Lab | Aspartate aminotransferase (AST or SGOT)_std | Variation of aspartate aminotransferase (AST or SGOT) | [–90, 0] |
| Lab | Basophil count (absolute count)_mean | Average value of basophil count (absolute count) | [–90, 0] |
| Lab | Basophil count (fraction)_mean | Average value of basophil count (fraction) | [–90, 0] |
| Lab | Bilirubin (Total), serum_mean | Average value of bilirubin (total), serum | [–90, 0] |
| Lab | Bilirubin (Total), serum_slope | Trend of bilirubin (total), serum | [–90, 0] |
| Lab | Bilirubin (Total), serum_std | Variation of bilirubin (total), serum | [–90, 0] |
| Lab | Calcium, serum_mean | Average value of calcium, serum | [–90, 0] |
| Lab | Calcium, serum_slope | Trend of calcium, serum | [–90, 0] |
| Lab | Calcium, serum_std | Variation of calcium, serum | [–90, 0] |
| Lab | Carbon dioxide_mean | Average value of carbon dioxide | [–90, 0] |
| Lab | Chloride_mean | Average value of chloride | [–90, 0] |
| Lab | Creatinine, serum_mean | Average value of creatinine, serum | [–90, 0] |
| Lab | Creatinine, serum_slope | Trend of creatinine, serum | [–90, 0] |
| Lab | Creatinine, serum_std | Variation of creatinine, serum | [–90, 0] |
| Lab | Eosinophils [volume] in Blood_mean | Average value of eosinophils [volume] in blood | [–90, 0] |
| Lab | Eosinophils/100 leukocytes in Blood_mean | Average value of eosinophils/100 leukocytes in blood | [–90, 0] |
| Lab | Glucose_mean | Average value of glucose | [–90, 0] |
| Lab | Glucose_slope | Trend of glucose | [–90, 0] |
| Lab | Glucose_std | Variation of glucose | [–90, 0] |
| Lab | Hematocrit_mean | Average value of hematocrit | [–90, 0] |
| Lab | Hematocrit_slope | Trend of hematocrit | [–90, 0] |
| Lab | Hematocrit_std | Variation of hematocrit | [–90, 0] |
| Lab | Hemoglobin, whole blood_count | Count of hemoglobin, white blood | [–90, 0] |
| Lab | Hemoglobin, whole blood_mean | Average value of hemoglobin, whole blood | [–90, 0] |
| Lab | Hemoglobin, whole blood_slope | Trend of hemoglobin, whole blood | [–90, 0] |
| Lab | Hemoglobin, whole blood_std | Variation of hemoglobin, whole blood | [–90, 0] |
| Lab | Lymphocyte Count (fractionated)_mean | Average value of lymphocyte count (fractionated) | [–90, 0] |
| Lab | Lymphocyte Count (fractionated)_slope | Trend of lymphocyte count (fractionated) | [–90, 0] |
| Lab | Lymphocyte Count (fractionated)_std | Variation of lymphocyte count (fractionated) | [–90, 0] |
| Lab | Lymphocyte Count Absolute_mean | Average value of lymphocyte count absolute | [–90, 0] |
| Lab | Lymphocyte Count Absolute_slope | Trend of lymphocyte count absolute | [–90, 0] |
| Lab | Lymphocyte Count Absolute_std | Variation of lymphocyte count absolute | [–90, 0] |
| Lab | Monocyte (fractionated)_mean | Average value of monocyte (fractionated) | [–90, 0] |
| Lab | Monocyte (fractionated)_slope | Trend of monocyte (fractionated) | [–90, 0] |
| Lab | Monocyte (fractionated)_std | Variation of monocyte (fractionated) | [–90, 0] |
| Lab | Monocyte count_mean | Average value of monocyte count | [–90, 0] |
| Lab | Monocyte count_slope | Trend of monocyte count | [–90, 0] |
| Lab | Monocyte count_std | Variation of monocyte count | [–90, 0] |
| Lab | Neutrophil count (absolute)_mean | Average value of neutrophil count (absolute) | [–90, 0] |
| Lab | Neutrophil count (absolute)_slope | Trend of neutrophil count (absolute) | [–90, 0] |
| Lab | Neutrophil count (absolute)_std | Variation of neutrophil count (absolute) | [–90, 0] |
| Lab | Platelet count_mean | Average value of platelet count | [–90, 0] |
| Lab | Platelet count_slope | Trend of platelet count | [–90, 0] |
| Lab | Platelet count_std | Variation of platelet count | [–90, 0] |
| Lab | Potassium [Moles/?volume] in Serum or Plasma_mean | Average value of potassium [moles/volume] in serum or plasma | [–90, 0] |
| Lab | Protein.Total, serum_mean | Average value of Protein.Total, serum | [–90, 0] |
| Lab | Protein.Total, serum_slope | Trend of Protein.Total, serum | [–90, 0] |
| Lab | Protein.Total, serum_std | Variation of Protein.Total, serum | [–90, 0] |
| Lab | Red Blood Cell Count_mean | Average value of red blood cell count | [–90, 0] |
| Lab | Red Blood Cell Count_slope | Trend of red blood cell count | [–90, 0] |
| Lab | Red Blood Cell Count_std | Variation of red blood cell count | [–90, 0] |
| Lab | Sodium [Moles/?volume] in Serum or Plasma_mean | Average value of sodium [moles/volume] in serum or plasma | [–90, 0] |
| Lab | Urea nitrogen_mean | Average value of urea nitrogen | [–90, 0] |
| Lab | Urea nitrogen_slope | Trend of urea nitrogen | [–90, 0] |
| Lab | Urea nitrogen_std | Variation of urea nitrogen | [–90, 0] |
| Lab | White Blood Cell Count_mean | Average value of white blood cell count | [–90, 0] |
| Lab | White Blood Cell Count_slope | Trend of white blood cell count | [–90, 0] |
| Lab | White Blood Cell Count_std | Variation of white blood cell count | [–90, 0] |
| Hist | advdxyr | Year of aNSCLC diagnosis | [–∞, 0] |
| Hist | daysfrom_ecogtoindex | Time from latest and highest ECOG score to index day | [–∞, 0] |
| Hist | ECOG_index | The latest and highest ECOG before or on index day | [–∞, 0] |
| Hist | initdxyr | Year of initial aNSCLC diagnosis | [–∞, 0] |
| Hist | n_admin | Number of admin visits before index date | [–90, 0] |
| Hist | n_diags | Number of diag visits before index date | [–90, 0] |
| Hist | n_ecog | Number of ECOG visits before index date | [–90, 0] |
| Hist | n_labs | Number of labs visits before index date | [–90, 0] |
| Hist | n_order | Number of order visits before index date | [–90, 0] |
| Hist | n_tele | Number of telemedicine visits before index date | [–90, 0] |
| Hist | n_vital | Number of vital visits before index date | [–90, 0] |
| Hist | smokingstatus_Non-smoker | Smoking status: non-smoker | [–∞, 0] |
| Hist | smokingstatus_Smoker | Smoking status: smoker | [–∞, 0] |
| Demo | age_index | Age at index day or LOT1 start day | NA |
| Demo | Sex | Sex: female 1, male 0 | NA |
| Demo | pCommercial | Commercial payer | [–∞, 0] |
| Demo | pMedicaid | Medicaid payer | [–∞, 0] |
| Demo | pMedicare | Medicare payer | [–∞, 0] |
| Demo | practicetype | Practice type | [–∞, 0] |
| Demo | race_BAA | Race: Black or African-American | NA |
| Demo | race_White | Race: White | NA |
| Demo | region_Midwest | Region: Midwest | [–∞, 0] |
| Demo | region_Northeast | Region: Northeast | [–∞, 0] |
| Demo | region_South | Region: South | [–∞, 0] |
| Demo | region_West | Region: West | [–∞, 0] |
| Como | ELX_GRP_1 | Congestive heart failure | [–∞, 0] |
| Como | ELX_GRP_10 | Chronic pulmonary disease | [–∞, 0] |
| Como | ELX_GRP_11 | Diabetes uncomplicated | [–∞, 0] |
| Como | ELX_GRP_12 | Diabetes complicated | [–∞, 0] |
| Como | ELX_GRP_13 | Hypothyroidism | [–∞, 0] |
| Como | ELX_GRP_14 | Renal failure | [–∞, 0] |
| Como | ELX_GRP_15 | Liver disease | [–∞, 0] |
| Como | ELX_GRP_16 | Peptic ulcer disease excluding bleeding | [–∞, 0] |
| Como | ELX_GRP_17 | AIDS/HIV | [–∞, 0] |
| Como | ELX_GRP_18 | Lymphoma | [–∞, 0] |
| Como | ELX_GRP_2 | Cardiac arrhythmia | [–∞, 0] |
| Como | ELX_GRP_21 | Rheumatoid arthritis/collagen | [–∞, 0] |
| Como | ELX_GRP_22 | Coagulopathy | [–∞, 0] |
| Como | ELX_GRP_23 | Obesity | [–∞, 0] |
| Como | ELX_GRP_24 | Weight loss | [–∞, 0] |
| Como | ELX_GRP_25 | Fluid and electrolyte disorders | [–∞, 0] |
| Como | ELX_GRP_26 | Blood loss anemia | [–∞, 0] |
| Como | ELX_GRP_27 | Deficiency anemia | [–∞, 0] |
| Como | ELX_GRP_28 | Alcohol abuse | [–∞, 0] |
| Como | ELX_GRP_29 | Drug abuse | [–∞, 0] |
| Como | ELX_GRP_3 | Valvular disease | [–∞, 0] |
| Como | ELX_GRP_30 | Psychoses | [–∞, 0] |
| Como | ELX_GRP_31 | Depression | [–∞, 0] |
| Como | ELX_GRP_4 | Pulmonary circulation disorders | [–∞, 0] |
| Como | ELX_GRP_5 | Peripheral vascular disorders | [–∞, 0] |
| Como | ELX_GRP_6 | Hypertension uncomplicated | [–∞, 0] |
| Como | ELX_GRP_7 | Hypertension complicated | [–∞, 0] |
| Como | ELX_GRP_8 | Paralysis | [–∞, 0] |
| Como | ELX_GRP_9 | Other neurological disorders | [–∞, 0] |
| Co-med | A01A | Stomatological preparations | [–90, 0] |
| Co-med | A02B | Drugs for peptic ulcer and gastroesophageal reflux disease | [–90, 0] |
| Co-med | A03C | Antispasmodics in combination with psycholeptics | [–90, 0] |
| Co-med | A03D | Antispasmodics in combination with analgesics | [–90, 0] |
| Co-med | A04A | Antiemetics and antinauseants | [–90, 0] |
| Co-med | A11C | Vitamin A and D, incl. combinations of the two | [–90, 0] |
| Co-med | B03A | Iron preparations | [–90, 0] |
| Co-med | B03B | Vitamin B12 and folic acid | [–90, 0] |
| Co-med | C02L | Antihypertensives and diuretics in combination | [–90, 0] |
| Co-med | C05A | Agents for treatment of hemorrhoids and anal fissures for topical use | [–90, 0] |
| Co-med | D07A | Corticosteroids, plain | [–90, 0] |
| Co-med | D07C | Corticosteroids, combinations with antibiotics | [–90, 0] |
| Co-med | D07X | Corticosteroids, other combinations. | [–90, 0] |
| Co-med | D08A | Antiseptics and disinfectants | [–90, 0] |
| Co-med | D10A | Anti-acne preparations for topical use | [–90, 0] |
| Co-med | H02A | Corticosteroids for systemic use, plain | [–∞, 0] |
| Co-med | L01B | Antimetabolites. | [–90, 0] |
| Co-med | L01C | Plant alkaloids and other natural products | [–90, 0] |
| Co-med | L01X | Other antineoplastic agents | [–90, 0] |
| Co-med | M01A | Anti-inflammatory and antirheumatic products, non-steroids | [–90, 0] |
| Co-med | N01B | Anesthetics, local | [–90, 0] |
| Co-med | N02A | Opioids | [–90, 0] |
| Co-med | N02B | Other analgesics and antipyretics | [–90, 0] |
| Co-med | N05A | Antipsychotics | [–90, 0] |
| Co-med | N05C | Hypnotics and sedatives | [–90, 0] |
| Co-med | N06C | Psycholeptics and psychoanaleptics in combination | [–90, 0] |
| Co-med | R01A | Decongestants and other nasal preparations for topical use | [–90, 0] |
| Co-med | R05D | Cough suppressants, excl. Combinations with expectorants | [–90, 0] |
| Co-med | S01B | Anti-inflammatory agents | [–90, 0] |
| Co-med | S01C | Anti-inflammatory agents and anti-infectives in combination | [–90, 0] |
| Co-med | S02B | Corticosteroids | [–90, 0] |
| Co-med | S02C | Corticosteroids and anti-infectives in combination | [–90, 0] |
| Co-med | S03B | Corticosteroids | [–90, 0] |
| Co-med | S03C | Corticosteroids and anti-infectives in combination | [–90, 0] |
| Co-med | V04C | Other diagnostic agents | [–90, 0] |

Como, comorbidities; Demo, demographics; Hist, medical history; Co-med, concomitant medications in ATC4 class; Trt, treatment; Lab, laboratory measurements; Vital, vital signs; Meta, metastasis; tumor, tumor characteristics; [-∞, 0], the entire time prior to or on index day; [-90, 0], 90 days prior to or on index day

**Table S2.** Hyperparameter tuning using randomized search of different parameter settings and 5-fold cross-validation for a) Overall survival and b) Progression-free survival. CPH, Cox proportional hazard; AFT, accelerated failure time; GBDT, gradient-boosted decision tree; SSVM, survival support vector machine.

a) Overall survival

| **model** | **parameter** | **parameter space** | **chosen value** |
| --- | --- | --- | --- |
| CPH | alpha | 10, 50, 100, 500, 1000 | 1000 |
|  | Ties | "breslow", "efron" | "breslow" |
|  | Max iteration | 100, 150, 200, 250, 300 | 100 |
|  | tol | 1e-9, 1e-7, 1e-5 | 0.00001 |
| LogLogisticAFT | Alpha | 0.001, 0.005, 0.01, 0.05, 0.1, 0.5 | 0.001 |
|  | Penalizer | 0, 0.01, 0.05, 0.1, 0.5 | 0 |
| GBDT (CoxPH) | No. trees | 100, 150, 200, 250, 300 | 250 |
|  | Max depth | 1, 2 | 2 |
|  | Learning rate | 0.005, 0.01, 0.05, 0.1 | 0.1 |
|  | Subsamples | 0.6, 0.7, 0.8 | 0.6 |
| SSVM | Alpha | 0.1, 0.5, 1, 5, 10 | 5 |
|  | Rank ratio | 0, 0.5, 1 | 0.5 |
|  | Max iteration | 10, 20, 30, 40, 50 | 30 |
|  | Optimizer | "avltree", "rbtree" | "avltree" |
| DeepSurv | Learning rate | 1e-4, 3e-4, 1e-3, 1e-2, 1e-1 | 0.01 |
|  | Dropout rate | 0.05, 0.10, 0.15, 0.20 | 0.2 |
|  | No. of hidden layers | 2, 3, 4 | 4 |
|  | No. of nodes in hidden Layers | [32, 16], [210, 110], [50, 50, 50], [210, 110, 10], [210, 100, 50, 10] | [210, 100, 50, 10] |

b) Progression-free survival

| **model** | **parameter** | **parameter space** | **chosen value** |
| --- | --- | --- | --- |
| CPH | Alpha | 10, 50, 100, 500, 1000 | 1000 |
|  | Ties | "breslow", "efron" | "efron" |
|  | Max iteration | 100, 150, 200, 250, 300 | 100 |
|  | tol | 1e-9, 1e-7, 1e-5 | 1.00E-09 |
| LogLogisticAFT | Alpha | 0.001, 0.005, 0.01, 0.05, 0.1, 0.5 | 0.001 |
|  | Penalizer | 0, 0.01, 0.05, 0.1, 0.5 | 0 |
| GBDT (CoxPH) | No. trees | 100, 150, 200, 250, 300 | 300 |
|  | Max depth | 1, 2 | 2 |
|  | Learning rate | 0.005, 0.01, 0.05, 0.1 | 0.05 |
|  | Subsamples | 0.6, 0.7, 0.8 | 0.6 |
| GBDT (Squared) | No. trees | 100, 150, 200, 250, 300 | 300 |
|  | Max depth | 1, 2 | 2 |
|  | Learning rate | 0.005, 0.01, 0.05, 0.1 | 0.05 |
|  | Subsamples | 0.6, 0.7, 0.8 | 0.6 |
| SSVM | Alpha | 0.1, 0.5, 1, 5, 10 | 1 |
|  | Rank ratio | 0, 0.5, 1 | 0.5 |
|  | Max iteration | 10, 20, 30, 40, 50 | 10 |
|  | Optimizer | "avltree", "rbtree" | "avltree" |
| DeepSurv | Learning rate | 1e-4, 3e-4, 1e-3, 1e-2, 1e-1 | 0.01 |
|  | Dropout rate | 0.05, 0.10, 0.15, 0.20 | 0.2 |
|  | No. of hidden layers | 2, 3, 4 | 3 |
|  | No. of nodes in hidden Layers | [32, 16], [210, 110], [50, 50, 50], [210, 110, 10], [210, 100, 50, 10] | [210, 110, 10] |

**Table S3** Distribution of values for top 20 significant predictors identified by gradient-boosted Cox proportional hazard model for differentiating high- and low-risk groups. ECOG, Eastern Cooperative Oncology Group; PD-L1, programmed cell death-ligand 1.

|  | **High risk group** | **Low risk group** |
| --- | --- | --- |
| **Overall survival** |  |  |
| ECOG performance status |  |  |
| 0 | 94 (11.9%) | 273 (34.7%) |
| 1 | 434 (55.1%) | 474 (60.3%) |
| 2 | 207 (26.3%) | 38 (4.8%) |
| 3 | 49 (6.2%) | 1 (0.1%) |
| 4 | 4 (0.5%) | 0 (0.0%) |
| Albumin serum mean | 2.55 (1.30) | 2.20 (1.05) |
| Body weight slope | -0.77 (1.70) | -0.04 (1.06) |
| PD-L1 percentage | 19.09 (31.78) | 28.30 (37.54) |
| Hematocrit mean | 36.44 (5.03) | 39.16 (4.02) |
| Cough suppressants, excluding combinations with expectorants | 0.55 (0.73) | 0.19 (0.49) |
| Chloride mean | 99.67 (3.56) | 101.28 (2.94) |
| Neutrophil per lymphocyte | 0.84 (1.38) | 0.36 (0.26) |
| Lymphocyte count (fractionated) mean | 15.16 (8.04) | 20.31 (7.50) |
| Alkaline phosphatase (ALP) mean | 113.85 (69.28) | 91.27 (27.75) |
| Age at index | 69.65 (9.42) | 68.74 (9.67) |
| Urea nitrogen mean | 18.04 (8.05) | 16.18 (5.82) |
| Albumin serum standard deviation | 2.55 (1.30) | 2.20 (1.05) |
| Albumin serum slope | -0.93 (1.99) | -0.42 (1.68) |
| White blood cell count mean | 10.62 (6.16) | 8.51 (2.91) |
| Metastasis site: liver/bile duct | 0.07 (0.25) | 0.02 (0.14) |
| Body weight standard deviation | 2.38 (2.03) | 1.85 (1.91) |
| Metastasis site: bone/bone marrow | 0.29 (0.45) | 0.13 (0.33) |
| Lymphocyte count (fractionated) slope | -1.79 (4.04) | -1.51 (4.70) |
| Monocyte count mean | 0.78 (0.41) | 0.65 (0.22) |
| **Progression-free survival** |  |  |
| PD-L1 percentage | 22.89 (32.97) | 31.64 (38.73) |
| Body weight slope | –0.83 (1.85) | –0.07 (1.05) |
| Lymphocyte count (fractionated) mean | 14.78 (7.69) | 20.46 (7.92) |
| ECOG performance status at index |  |  |
| 0 | 97 (16.3%) | 231 (34.7%) |
| 1 | 352 (59.2%) | 390 (58.6%) |
| 2 | 118 (19.8%) | 41 (6.2%) |
| 3 | 28 (4.7%) | 4 (0.6%) |
| 4 | 0 (0.0%) | 0 (0.0%) |
| Albumin serum mean | 36.22 (4.51) | 39.04 (3.81) |
| Alkaline phosphatase (ALP) mean | 115.88 (73.10) | 91.54 (33.28) |
| Chloride mean | 99.60 (3.66) | 101.24 (2.60) |
| Metastasis site: bone/bone marrow | 0.34 (0.47) | 0.09 (0.28) |
| Neutrophil per lymphocyte | 0.82 (1.11) | 0.39 (0.36) |
| Number of medication orders | 3.92 (3.66) | 2.46 (1.56) |
| Urea nitrogen mean | 18.67 (8.70) | 16.73 (6.44) |
| Cough suppressants, excluding combinations with expectorants | 0.61 (0.79) | 0.16 (0.41) |
| Hematocrit mean | 36.61 (5.25) | 39.26 (4.24) |
| Lymphocyte count (fractionated) slope | –1.94 (3.79) | –1.46 (4.39) |
| Protein total serum mean | 68.78 (6.08) | 70.03 (5.16) |
| Lymphocyte count (fractionated) standard deviation | 5.14 (3.09) | 5.31 (3.02) |
| Body weight standard deviation | 2.29 (1.97) | 1.70 (1.62) |
| Total bilirubin serum mean | 0.49 (0.24) | 0.47 (0.18) |
| ICI and chemotherapy |  |  |
| 0 | 266 (44.7%) | 280 (42.0%) |
| 1 | 329 (55.3%) | 386 (58.0%) |
| Albumin serum slope | –1.11 (1.97) | –0.42 (1.72) |
